# Linking sports registration information and player feedback to enhance netball participation

**DOI:** 10.1186/s13102-021-00286-0

**Published:** 2021-06-09

**Authors:** Bridget C. Foley, Catriona Rose, Katherine B. Owen, Lindsey J. Reece

**Affiliations:** The University of Sydney, Faculty of Medicine and Health, Sydney School of Public Health, Charles Perkins Centre, Prevention Research Collaboration, SPRINTER, Sydney, Australia

**Keywords:** Exercise, Sports, Recreation, Netball, Women

## Abstract

**Background:**

Sports should endeavour to provide inclusive opportunities for all people to participate. More evidence is required to understand who joins sports clubs and what keeps participants engaged throughout their lifetime. Little is known about who plays netball or what drives participation and retention of players in netball. This study aimed to identify the sociodemographic characteristics of Netball New South Wales (NSW) members, their odds of re-registering in the sport, and explore their motivations to play, experiences during participation and the perceived benefits of playing netball.

**Methods:**

We used longitudinal sport registration data from all Netball NSW members in 2018 and 2019. A cross-sectional online survey was sent to all players registered during the two-year study period (*n* = 157,152). We used logistic regression to determine the odds of individuals returning to netball in 2019 after playing in 2018 and linked the sports registration data with the survey responses, calculating frequencies and proportions.

**Results:**

Netball NSW members were mostly female (98 %) and aged less than 18 years old (69 %). Netball NSW retained 68 % of members in 2019 who played the previous year. Members who were male, aged 18–44 years old, lived in low SES areas, lived in regional/remote locations, identified as Aboriginal or Torres Strait Islander, spoke a language other than English at home or were born outside Australia had lower odds of returning to play from 2018 to 2019. Survey participants (*n* = 10,795) rated their experience playing netball highly and reported that playing netball improved their health and wellbeing. The main reason to play netball reported was ‘fun and enjoyment’ while the main reason to consider quitting was the ‘skill/experience of umpires and officials’.

**Conclusions:**

This study highlights the strengths of netball in engaging and retaining females, who often participate in less sport than males. The positive experiences reported by netball participants should be fostered to retain current participants throughout the lifecourse. The data provided by members should be inform strategic actions to enhance netball participation for sociodemographic groups who had greatest odds of dropout. Routine surveillance using linked registration and player feedback should be utilised by sports to enhance sport delivery and increase participation.

## Background

Participation in sport throughout life has many health, social and economic benefits for individuals and communities [1–3]. Sport participation is strongly associated with attainment of physical activity guidelines, development of physical literacy, prevention and management of chronic disease and is associated with positive physical and mental health [1, 4–6]. Opportunities to access sports activities are unequal in society, depending on peoples’ sex, age, location, cultural background, socioeconomic status, prior health and life experiences [2]. To deliver the benefits of sport participation and increase population physical activity levels, sports organizations and funders should use evidence to prioritise engagement of people from all socio-demographic groups and foster life-long participation [2].

Sport retention in this paper is defined as a participant *returning to play the same activity year after year*. When sport retention is lower than expected in a sports organisation, it suggests participants are either sampling different sport opportunities or have stopped sport participation altogether. Crawford and Godbey theorized many factors which influence sport retention, broadly categorized as intrapersonal, interpersonal and structural barriers [7, 8]. Monitoring retention rates to understand trends in participation should be an essential strategic focus for sports organizations [4]. Balish et al., estimates 70 % of youth club participants return to play their sport each year [9, 10]. Australian research suggests that only 45 % of sport participants aged 5–14 years old are retained across multiple sports; and that retention rates halve for 15–19 year old participants and continue to decline with age [11]. To strengthen life-long sport participation, evidence suggests it is beneficial for children to sample many sports (potentially producing lower retention rates during childhood) rather than specialise in a particular sport [11]. Promotion of sport sampling in younger age groups and ensuring fun and enjoyment in activities throughout the sport sector is likely to lead to greater retention of sport participants throughout the life-course [11]. Although improving sport retention is internationally important, sport retention rates for children or adults are not discussed within current literature [10].

Women and girls are less physically active than men and boys globally, and participate in less sport [12–14]. Gender-specific action to reduce the prevalence of physical inactivity in women and girls, without changes to the prevalence in men and boys, would achieve the 2025 World Health Organisation target for inactivity [12]. In Australia, netball is a popular team sport for women and girls who dominate participation, with increasing opportunities for men and boys [13]. Netball is played in school, community, and state competitions at grassroots, representative and elite levels. Participation in netball at all levels requires moderate-vigorous physical activity, catching, throwing, coordination and teamwork, among other skills. National surveillance data provides high-level data from netball players [13], however detailed understanding of netball participation over time and the impacts playing netball has on people lives is lacking.

Utilization of sports membership data can supplement population estimates and provide unique insights regarding sport retention [15]. Sports organisations can develop further insights through appropriate primary data collection to inform sport-specific actions to increase participation and retention of players. As a sport dominated by women and girls, exploration of netball participation may provide insights towards reducing the prevalence of inactivity in women and girls through sport. The purpose of this study was to identify trends in netball participation and retention to monitor community engagement over a two-year period. This study aims to identify socio-demographic groups which are under-represented in netball and explore factors which are associated with netball retention using membership data from Netball New South Wales (NSW) – the peak state sporting organisation responsible for the governance, development, promotion and administration of netball throughout NSW, affiliated to Netball Australia.

## Methods

This descriptive study utilized longitudinal, routinely collected membership data from Netball NSW and primary data collected through a cross-sectional online survey. The University of Sydney Human Research Ethics Committee granted ethics approval for this study (Protocol number: 2019/906).

### Membership data

Netball NSW provided complete membership data extracted from their member registration platform for this study. All individuals who held a Netball NSW membership in 2018 or 2019 were included. Membership data included unique member identification code, sex, date of birth, postcode, Aboriginal identity, disability status, language spoken at home and country of birth. The unique member identification code was used to match members in the 2019 dataset with those who were members in 2018, to ascertain member retention. Postcode of residence was used to determine socioeconomic status (Socio-Economic Indexes for Areas Index of Relative Disadvantage [16]) and location (Accessibility/Remoteness Index of Australia [17]). Using self-reported height and weight, Body Mass Index was calculated using International Obesity Task Force cut points [18].

Membership data also included a dichotomous variable for Active Kids voucher use. At the time of the study, a universal voucher program entitled Active Kids was being implemented by the state Government which aimed to reduce the cost of registration in sport and recreational physical activities for all school-enrolled children in NSW [19]. In 2018, more than half the NSW population registered for an Active Kids voucher to reduce the cost of netball [20]. Therefore, Active Kids voucher use was included as a covariate in this study.

### Online survey

**Fig. 1:**
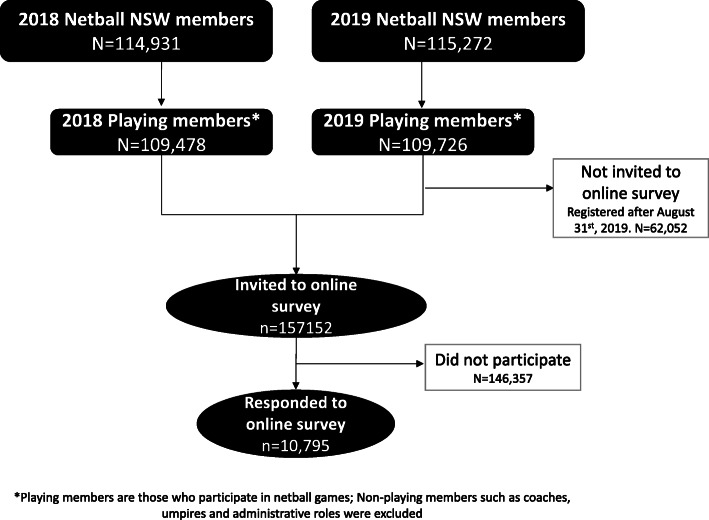
Participant recruitment flow diagram

Members who registered between January 1st, 2018 and August 31st, 2019 were also invited to voluntarily participate in an online survey during December 2019 (Fig. 1). Netball NSW members were emailed the study participant information sheet and survey link, with a reminder email sent after two weeks. The survey was designed to be completed independently by netball players who were over the age of 16 years old or by children with the support of their parent/carer or an appropriate adult. Members who participated in the survey were entered in a prize draw.

The survey included questions created specifically for this study which asked members about their motivations for playing, perceived benefits of participation and asked them to rate their experience of different aspects of netball. Participants also reported their height, weight and chronic health conditions. A full copy of the survey is available from the authors, upon reasonable request. International Physical Activity questionnaire (IPAQ) items were used to understand their physical activity levels in the last 7 days [21]. Physical activity levels were defined as High, Moderate or Low level of physical activity using the IPAQ scoring protocol [22]. Scoring a high level equates to approximately ≥ 1-hour of moderate to vigorous intensity activity per/day, moderate level equates to 30-minutes of at least moderate intensity activity on most days, and low level is not achieving either high or moderate level of physical activity [22]. Those with low levels physical activity are not meting physical activity guidelines [23]. AusPlay items were used to measure sport participation (number of sessions per year), annual expenditure on sport, and whether the member was considering giving up netball [24].

### Data analysis

Survey responses were downloaded from the survey platform (Qualtrics, Version Dec 2019. Provo, UT, USA. https://www.qualtrics.com) and linked with membership registration data using the unique member identification code. All data analysis was completed using Statistical Package for the Social Sciences (IBM SPSS Statistics, Version 25. Armonk, NY: IBM Corp). Where appropriate, adult and child outcomes have been analyzed separately.

To understand the characteristics of participants each year we calculated descriptive statistics including frequency and proportions and assessed for significant differences between years using Pearson Chi-Squared tests (Significance level *P* < 0.05). Next, we used logistic regression to determine the odds ratio (OR) for a player returning to netball in 2019 after playing in 2018 with 95 % confidence intervals (CI). We calculated descriptive statistics for the survey responses and assessed significant differences using Pearson Chi-Squared tests (Significance level *P* < 0.05) between those considering quitting netball or who had already quit in 2019, compared to those planning to continue to play netball in 2020.

## Results

### Netball NSW Registration data

The total number of netball players registered with Netball NSW remained similar from 2018 to 2019. Retention of players from 2018 to 2019 was 68 %, with 32 % (*n* = 35,016) of 2018 members not renewing their membership after playing in 2018. Members who were male, aged 18–34 years old, lived in low SES areas, lived in regional/remote locations, spoke a language other than English at home or were born outside Australia had lower odds of returning to play from 2018 to 2019 (Table 1). Playing members had a median age of 14 years old (Interquartile range [IQR] = 10) with 70 % of all Netball NSW members aged less than 18 years old. Members who joined Netball NSW in 2019 had a median age of 10 years (IQR = 5) in children and 28 years (IRQ = 15) in adults. For male Netball NSW members (Median age 23, IRQ = 15) retention was 33 % from 2018 to 2019.

**Table 1:** Netball NSW membership sociodemographic characteristics with odds ratios

Demographic characteristic		2018 Playing members (*n*=109,478)		2019 Playing members (*n*=109,726)		Odds of retaining 2018 members in 2019 (*n*=109,478)	
		n	%	n	%	Odds Ratio (95% CI)	*P* value
Sex	Female	107226	97.9	106956	97.5	Ref	
	Male	2197	2.0	2732	2.5	0.2 (0.2, 0.2)	<0.0001
Age category	5-6 years	2968	2.7	2703	2.5	Ref	
	7-9 years	17337	15.8	16917	15.4	2.3 (2.1, 2.5)	<0.0001
	10-12 years	24969	22.8	24669	22.5	2.3 (2.2, 2.5)	<0.0001
	13-14 years	14922	13.6	15611	14.2	1.9 (1.8, 2.1)	<0.0001
	15-17 years	15915	14.5	15860	14.5	1.1 (1.0, 1.2)	0.056
	18-24 years	12510	11.4	12634	11.5	0.7 (0.6, 0.7)	<0.0001
	25-34 years	10033	9.2	10146	9.2	0.7 (0.7, 0.8)	<0.0001
	35-44 years	7248	6.6	7397	6.7	0.9 (0.8, 0.9)	0.001
	45-54 years	3127	2.9	3283	3.0	0.9 (0.8, 1.0)	0.005
	55-64 years	426	0.4	475	0.4	1.1 (0.9, 1.3)	0.528
	65+ years	5	0.0	8	0.0	0.8 (0.4, 1.9)	0.638
Socio economic status (SES)	Forth quartile (High SES)	41534	37.9	41765	38.1	Ref	
	Third quartile	26428	24.1	26516	24.2	0.9 (0.9, 0.9)	<0.0001
	Second quartile	26618	24.3	26815	24.4	0.7 (0.7, 0.7)	<0.0001
	First quartile (Low SES)	13996	12.8	13561	12.4	0.6 (0.5, 0.6)	<0.0001
	Missing	902	0.8	1069	1.0	-	
Location	Major Cities	73293	66.9	73399	66.9	Ref	
	Inner Regional	26417	24.1	26535	24.2	0.7 (0.7, 0.7)	<0.0001
	Outer Regional/ Remote	8458	7.7	8255	7.5	0.5 (0.5, 0.6)	<0.0001
	Missing	1310	1.2	1537	1.4	-	
Identifies as Aboriginal/ Torres Strait Islander	No	95900	87.6	98263	89.6	Ref	
	Yes	5922	5.4	6577	6.0	0.7 (0.6, 0.7)	<0.0001
	Undisclosed	6780	6.2	3969	3.6		
Disability status	No	99790	91.2	103619	94.4	Ref	
	Yes	720	0.7	770	0.7	0.9 (0.7, 1.0)	0.053
	Undisclosed	8047	7.4	4373	4.0	-	
Language spoken at home	English only	91545	83.6	95253	86.8	Ref	
	Language other than English	6732	6.1	7533	6.9	0.9 (0.8, 0.9)	<0.0001
	Undisclosed	10172	9.3	5873	5.4	-	
Country of birth	Australia	98942	90.4	100610	91.7	Ref	
	Outside Australia	10536	9.6	9116	8.3	0.6 (0.6, 0.6)	<0.0001

There were significant differences (*P*<0.05) between 2018 and 2019 members for all demographic characteristics, except socio-economic status (*P*=0.053). Undisclosed was treated as missing in the analysis

*Ref* Reference category, *CI* Confidence Interval

In 2018, 67 % of all eligible Netball NSW members used an Active Kids voucher to subsidize the cost of their participation. Retention of players from 2018 to 2019 was twice as likely if they used an Active Kids voucher in 2018 (OR 1.9 [95 % CI 1.8, 1.9]). In 2019, the proportion who used a voucher increased to 72 % of eligible members using an Active Kids voucher.

### Survey responses

Of the members invited to participate in the research, 10,795 (6.9 %) responded to the online survey (Table 2). Additional demographic information gained showed that 14 % of participants reported living with an ongoing physical health condition, most commonly a muscular skeletal condition (7 %) or respiratory condition such as Asthma (2 %). Most participants were categorized as having a healthy weight 58 % while 8 % were categorized as thin, 22 % overweight and 13 % obese.

**Table 2:** Characteristics of survey participants compared to invited Netball NSW members

		Invited *n*=157,152		Participated *n*=10,795		Response rate 6.9%
		n	%	n	%	%
Sex	Female	149461	95.1	10312	95.5	7.6
	Male	3106	2.0	144	1.3	4.8
Age category	5-6 years	3041	1.9	223	2.1	7.4
	7-9 years	20477	13.0	1725	16.0	8.6
	10-12 years	31712	20.2	2902	26.9	9.9
	13-14 years	21618	13.8	2491	23.1	9.1
	15-17 years	24318	15.5	623	5.8	4.6
	18-24 years	21512	13.7	963	8.9	4.9
	25-34 years	16639	10.6	877	8.1	5.9
	35-44 years	11639	7.4	558	5.2	5.3
	45-54 years	5367	3.4	318	2.9	6.7
	55-64 years	782	0.5	109	1.0	16.2
	65+ years	47	0.0	6	0.1	14.6
Socio economic status (SES)	First quartile (Low SES)	23846	15.2	1381	12.8	6.3
	Second quartile	43095	27.4	2816	26.1	7.1
	Third quartile	25149	16.0	1982	18.4	8.5
	Forth quartile (High SES)	59243	37.7	4197	38.9	7.9
	Missing	5819	3.7	419	3.9	7.2
Location	Major Cities	92629	58.9	7436	68.9	8.0
	Inner Regional	34008	21.6	2302	21.3	6.8
	Outer Regional and Remote	11204	7.1	648	6.0	5.8
	Undisclosed	19311	12.3	409	3.8	2.1
Identifies as Aboriginal/ Torres Strait Islander	No	129887	82.7	9452	87.6	7.7
	Yes	8721	5.5	509	4.7	6.2
	Undisclosed	18544	11.8	834	7.7	4.5
Disability status	No	136232	86.7	9789	90.7	7.6
	Yes	991	0.6	80	0.7	8.5
	Undisclosed	19929	12.7	926	8.6	4.6
Language spoken at home	English only	124536	79.2	8921	82.6	7.6
	Language other than English	9919	6.3	825	7.6	8.8
	Undisclosed	22697	14.4	1049	9.7	4.6
Country of birth	Australia	126016	80.2	9505	88.1	7.5
	Outside Australia	13591	8.6	954	8.8	7.0
Body Mass Index	Thin	NA	NA	535	5.0	NA
	Healthy weight	NA	NA	4010	37.1	NA
	Overweight	NA	NA	1509	14.0	NA
	Obese	NA	NA	924	8.6	NA
	Not reported	NA	NA	3817	35.4	NA
Chronic Health Condition	No	NA	NA	6497	60.2	NA
	Yes	NA	NA	1095	10.1	NA
	Undisclosed			3203	29.7	
Mental Health	Anxiety	NA	NA	1128	10.4	NA
	Depression	NA	NA	549	5.1	NA
	Other	NA	NA	176	1.6	NA
	None	NA	NA	6044	56.0	NA
	Undisclosed			2898	26.8	

*NA* not available

#### Contribution of netball to physical activity participation

Netball sessions made up approximately half (median 53 %) of all participation in recreational physical activity per year. Members reported netball participation lasted 60 min per week (Standard deviation = 57) during the netball season. Netball NSW playing members had high/moderate levels of physical activity with 75 % of adults and 82 % of children meeting the Australian Physical Activity guidelines for good health [23]. About half the survey participants could recall the recommended amount of physical activity adults and children should achieve each week (58 % recalled adult guideline; 48 % recalled child guidelines) [23].

#### Impacts of netball

The perceived personal and social impacts of playing netball for both adults and children were reported in this study and overall, playing netball had positive impacts on participants’ lives. Adults perceived that netball increased their social connection (74 %), strength/fitness (70 %), overall health improvements (67 %), skills and coordination (66 %) and energy levels (65 %) while less than 2 % reported a decrease in these areas. Furthermore, adults reported that playing netball decreased their feelings of anxiety (41 %) or they stayed about the same (50 %), and netball decreased their number of sick days from work (18 %) or they stayed about the same (78 %). These impacts were similar whether the respondent was planning to continue or considering quitting netball.

For children, highly positive impacts of netball including increased skills and coordination (88 %), strength and fitness (78 %), ability to work in a team (77 %), social connection (73 %) and self-confidence (68 %) while less than 3 % of children perceived a decrease in these areas. Participants reported that playing netball decreased children’s feelings of anxiety (32 %) or they stayed about the same (56 %), and playing netball decreased children’s number of sick days from school (15 %) or they stayed about the same (80 %). Among children who were considering quitting netball, 12 % reported that netball decreased their self-confidence, compared to 4 % of those planning to continue netball.

#### Quality of the player experience

**Fig. 2:**
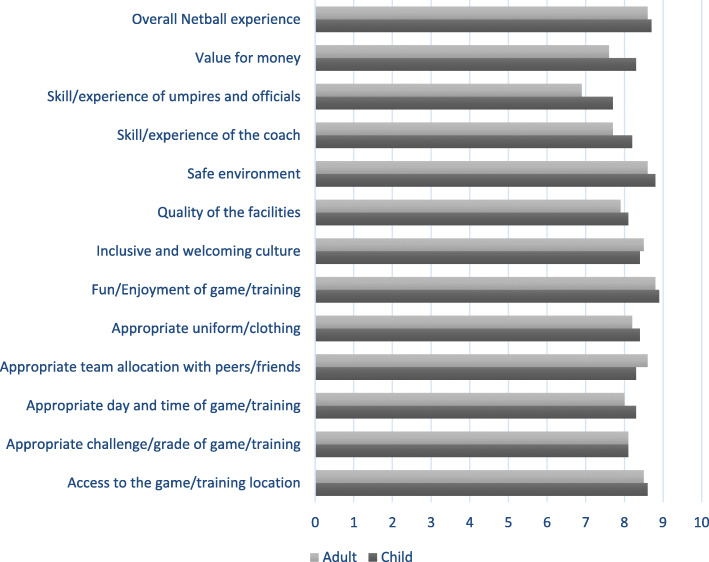
Average ratings of netball participation through a Club/Association, where 0 is Poor and 10 is Excellent

Participants were asked to rate their experience in netball between 0 and 10, where a rating of 0 is poor and 10 is excellent. On average, respondents rated their overall experience playing netball 8.7 out of 10 (*Standard deviation* = 1.5). The ‘skill/experience of umpire and officials’ received the poorest rating for both adults and children, 6.9 and 7.7 out of 10, respectively (Fig. 2). Most respondents (74 %) were planning to continue playing netball in the next 12 months; the remaining 26 % of respondents were considering quitting or had already quit. One in three (36 %) adults reported ‘injury or fear of injury’ as their main reason for giving up netball. The main reasons reported by respondents under 18 years old were ‘Not fun or enjoyable anymore’ (15 %) and/or they had a ‘Bad experience with coach/official’ (14 %).

#### Netball retention factors

Sociodemographic factors which influence retention from 2018 to 2019 are described earlier. Survey participants reported whether they were considering quitting netball in the next 12 months or had already quit netball. Those who identified they were considering quitting or had already quit netball were significantly less likely to meet physical activity guidelines (Adults *p* < 0.001, Children *p* < 0.001). Adults were also more likely to quit netball if they had a chronic health condition. Children who were male (p 0.017), teenagers (13 + year old) (*p* < 0.001), outside the health weight range (*p* 0.031), or had a diagnosed mental health condition (*p* < 0.001) were significantly more likely to be consider quitting or had already quit netball.

When asked about their main reasons to play netball, 61 % of participants reported playing ‘For fun/enjoyment’, followed by the social motivation of ‘being part of a team’ which remained relevant for both adults (41 %) and children (52 %). In addition, common reasons adults participated in netball were improving fitness and performance (39 %), to play competitively (36 %) and improving health and preventing illness (32 %); whilst children’s reasons included spending time with friends (36 %), to improve fitness/performance (32 %), to play competitively (31 %) and improve skills and coordination (30 %).

## Discussion

This study utilized existing membership data and collected new outcome focused information from Netball NSW playing members. The analysis of two consecutive years of Netball NSW memberships provides the first exploration of a sample larger than 2,000 netball players [13], with over 100,000 players included for both 2018 and 2019. Of these, 74,462 (68 %) were a member in both years. Most netball players were female and under 18 years old, which is consistent with the National sport surveillance, AusPlay [13]. Survey respondents rated their overall experience playing netball highly, which was reflected in the high retention of women and girls from year to year. There was high representation of people who identify as Aboriginal and Torres Strait Islander (5 % in 2018 and 6 % in 2019 memberships) compared to the NSW population (3.6 %) [25]. People who spoke a language other than English at home, were physically inactive, male, and socio-economically disadvantaged were underrepresented among the netball community compared to NSW population surveillance [26, 27]. The benefits of netball participation reported by players in this study suggests opportunities for netball participation in NSW should be extended. The netball community should strive to engage more culturally and linguistically diverse players, physically inactive women (as this sport is highly acceptable for women and girls), men, and socioeconomically disadvantaged players.

The outcome and sport participation data collected in this study is not typically collected or reported by sports organisations [10]. This study collected primary research data which provides new insights into sport participation, player’s physical activity levels and health related outcomes. Results indicate that playing netball constituted more than half of participants’ annual sessions of sport. The positive impacts players associated with netball participation reflected priorities for participation in different life stages; for example most adults reported that netball increased their social connection, which often declines with age, while children and their guardians reported positive impacts on skill development as they set the foundations of their physical literacy during this life stage [1, 8]. Previous research has shown that understanding women and girls’ motivations for sport participation can guide successful program design and increase retention [1, 11, 28]. A systematic review investigating why children stop playing sport found that lack of enjoyment, perceptions of competence, social pressures, competing priorities and physical factors (maturation and injuries) were the main reasons [8]. These reasons were consistent with the children in our study. In contrast, one third of adults were considering quitting netball because they had a fear of injury. The most common netball injuries are to the knees and ankles [29] and these appear to be increasing in prevalence [30]. Although sports medicine injury prevention programs for netball are known and established, their translation into courtside practice at all levels of the sport appear limited [29]. Further research into effective implementation of injury prevention programs should be undertaken to minimise injury occurrence, and individuals fear of injury when playing netball. Our study found that netball participation had no impact or reduced the quantity of sick days taken. This finding implies the need to communicate that the benefits of playing may outweigh the risks. The positive impacts of playing netball, which counter perceptions behind considering quitting, should be highlighted to current and potential new members to increase participation.

Successful delivery of sports programs requires skilled, enthusiastic individuals within sports organizations [31]. Our study found that both adults and children rated the skills/experience of umpires and officials lower than any other aspect of netball. Netball Australia has an umpire development framework however these results suggest improvement in this area may aid retention. Netball has distinctive rules that define the skills of the game and the limits within which they must be performed. For example, a player can hold the ball for a maximum of three seconds, take less than two steps with ball possession, and only two players in each team may enter the goal circle to take a shot at goal. As a result, the skills and experience of umpires and officials can influence the player experience. Initiatives which enhance the skill and experience of people involved in the delivery of the sport at grassroots is known to enhance the player experience [28, 32]. In New Zealand, umpire development pathways which utilize incentives and accreditations within the pathway have been found to increase recruitment, however retention of skilled and experienced umpires and officials in grass-roots netball remained limited [33]. Netball and similar sports organisations should ensure that those in non-player roles involved in the delivering a quality sport product are supported to facilitate positive experiences for sport participants.

To increase participation in sport and physical activity a sport-for-all approach should be adopted, prioritising underrepresented groups in sports organisations recruitment and retention goals [27]. Identification of member’s who are considering quitting and intervening to reduce drop out is critical [15]. Adaptions from the existing model of sport delivery may be required to engage underrepresented groups. Best practice approaches to developing appropriate modified programs adopting a sport for all approach include meaningful engagement with the target group, pilot testing, refinement, and efficacy testing before scaling these up [34]. In the United Kingdom, a program entitled ‘Back to Netball’ has been designed to re-introduce adult women to playing netball [28]. ‘Back to Netball’ found that social factors such as connection with team mates and competence development motivated women to participate in netball [28]. Motivations and perceived benefits of participation in netball should be combined with behaviour change theories to inform strategies which appeal to the values of prospective members. Sports organisations face a range of challenges when trying to develop and deliver innovative modified sport products, but capacity within the sport sector needs to be enhanced to prioritise engagement and retention of priority, underrepresented groups [32].

Player characteristics associated with considering quitting netball identified in this study, are consistent with societal public health issues [2]. Addressing physical inactivity, overweight and obesity, and mental health conditions associated with quitting netball requires collaboration with other sectors such as health, education and transport to overcome barriers to participation. The NSW Government Office of Sport has trialled the implementation of financial incentives for children to reduce the barrier of cost to sport participation [19]. The present study found that this large-scale government intervention (Active Kids) significantly increased short-term retention of children in netball. Additional strategies in collaboration with other sectors may include establishing a partnership between the sport organization and youth mental health services. These mutually beneficial interventions may have benefits for sport retention and reduce mental illness in the community. Netball players who are not meeting physical activity guidelines are an ideal group for targeted physical inactivity interventions [31, 32].

Due to the recent introduction of an online membership platform, Netball NSW was able to provide two years of reliable, comparable membership data for this study. With improvements in membership platforms and standardized data collection, there is potential to monitor sport retention regularly. Longitudinal linked data from netball NSW members which allow retention to be investigated over multiple years would provide deeper understanding of member retention. The methods adopted for this study should be continued annually to monitor improvements in participation and member retention in netball and other sports. Findings from this study can also help inform other sport, recreation and physical activity providers who are interested in growing women and girls participation in their sport. Sports organizations need to invest in placing members at the heart of their operations in order to respond to changing needs and participation trends. Caution must be taken when inferring from this data, as members participating in the online survey (6.9 % of invited members) were highly engaged in netball and this bias should be considered before these results are generalized to all Netball NSW members or the general population. We note that this study does not represent all individuals who play netball in NSW as people may play in other community settings, not associated with Netball NSW. Furthermore, this study did not investigate why non-players (coaches, umpires, officials, committee members) participate in netball; these members should be considered in future to understand participation across the whole sports organisation.

## Conclusions

Netball engages many women and girls in sport each year. Participants reported that playing netball contributed to their overall participation in physical activity, improved skills and competencies, social connection and improved their health and wellbeing. There is a need to enhance capability and capacity of clubs and non-player members such as umpires and officials, to improve retention of engaged players into adulthood. Alternative strategies that attract underrepresented groups and those considering quitting netball must be prioritised. Consistent monitoring of netball participants with in-depth data collected on the outcomes associated with participation in netball could be integrated into routine data capture. Improved use of locally relevant netball data will help enhance the quality of the player experience and enable evidence-based decision making to grow participation and increase retention.

## Data Availability

The data that support the findings of this study are available from the corresponding author, BCF, upon reasonable request.

## Abbreviations

NSW: New South Wales
IPAQ: International Physical Activity Questionnaire

## References

[1] Eime RM, Young JA, Harvey JT, Charity MJ, Payne WR (2013). A systematic review of the psychological and social benefits of participation in sport for adults: informing development of a conceptual model of health through sport. Int J Behav Nutr Phys Act.

[2] Wolrd Health Organization (2018). Global action plan on physical activity 2018–2030: more active people for a healthier world.

[3] Oja P, Titze S, Kokko S, Kujala UM, Heinonen A, Kelly P (2015). Health benefits of different sport disciplines for adults: systematic review of observational and intervention studies with meta-analysis. Br J Sports Med.

[4] Palomaki S, Hirvensalo M, Smith K, Raitakari O, Mannisto S, Hutri-Kahonen N (2018). Does organized sport participation during youth predict healthy habits in adulthood? A 28-year longitudinal study. Scand J Med Sci Sports.

[5] Telford RM, Telford RD, Cochrane T, Cunningham RB, Olive LS, Davey R (2016). The influence of sport club participation on physical activity, fitness and body fat during childhood and adolescence: The LOOK Longitudinal Study. J Sci Med Sport.

[6] Vella SA, Schranz NK, Davern M, Hardy LL, Hills AP, Morgan PJ (2016). The contribution of organised sports to physical activity in Australia: Results and directions from the Active Healthy Kids Australia 2014 Report Card on physical activity for children and young people. J Sci Med Sport.

[7] Crawford DW, Godbey G (1987). Reconceptualizing barriers to family leisure. Leisure Sci.

[8] Crane J, Temple V (2014). A systematic review of dropout from organized sport among children and youth. Eur Phys Educ Rev.

[9] Balish SM, McLaren C, Rainham D, Blanchard C (2014). Correlates of youth sport attrition: A review and future directions. Psychol Sport Exer.

[10] Howie EK, Guagliano JM, Milton K, Vella SA, Gomersall SR, Kolbe-Alexander TL (2020). Ten Research Priorities Related to Youth Sport, Physical Activity, and Health. J Phys Act Health.

[11] Eime RM, Harvey JT, Charity MJ (2019). Sport drop-out during adolescence: is it real, or an artefact of sampling behaviour?. Int J Sport Policy Polit.

[12] Mielke GI, da Silva ICM, Kolbe-Alexander TL, Brown WJ (2018). Shifting the Physical Inactivity Curve Worldwide by Closing the Gender Gap. Sports Med.

[13] Sport Australia. AusPlay Results: Clearinghouse for Sport; 2020 [Available from: https://www.clearinghouseforsport.gov.au/research/smi/ausplay/results.

[14] Guthold R, Stevens GA, Riley LM, Bull FC (2018). Worldwide trends in insufficient physical activity from 2001 to 2016: a pooled analysis of 358 population-based surveys with 1·9 million participants. Lancet Global Health.

[15] Eime RM, Harvey JT, Charity MJ, Casey MM, Westerbeek H, Payne WR (2016). Age profiles of sport participants. BMC Sports Science, Medicine and Rehabilitation.

[16] Australian Bureau of Statistics (2006). Census of population and housing: Socio-Economic Indexes for Areas (SEIFA) - Technical paper.

[17] Australian Department of Health and Aged Care (2001). Measuring remoteness: Accessibility/Remoteness Index of Australia (ARIA).

[18] Cole TJ, Lobstein T (2012). Extended international (IOTF) body mass index cut-offs for thinness, overweight and obesity. Pediatr Obes.

[19] Reece LJ, Foley BC, Bellew B, Owen K, Cushway D, Srinivasan N, Hamdorf P, Bauman AE. Active kids; evaluation protocol for a universal voucher program to increase children’s participation in organised physical activity and sport. Public Health Res Pract. 2020. 10.17061/phrp30122006. 10.17061/phrp30122006 34104931

[20] Foley BC OK, Bellew W, Wolfenden L, Reilly K, Bauman AE, Reece LJ. Physical activity behaviors of children who register for the universal, state-wide active kids voucher: Who did the voucher program reach? Int J Environ Res Public Health. 2020;17(16):5691. 10.3390/ijerph17165691. 10.3390/ijerph17165691 PMC7460334 32781753

[21] Milton K, Bull FC, Bauman A (2011). Reliability and validity testing of a single-item physical activity measure. British journal of sports medicine.

[22] International Physical Activity Questionnaire. Guidelines for Data Processing and Analysis of the International Physical Activity Questionnaire (IPAQ) – Short and Long Forms. 2005.

[23] Australian Government, Department of Health. Physical activity and exercise guidelines for all Australians. Canberra: Commonwealth Department of Health. 2019. Available: https://www.health.gov.au/health-topics/physical-activity-and-exercise/physical-activity-and-exercise-guidelines-for-all-australians.

[24] Australian Sports Commission (2016). AusPlay: Survey Questions.

[25] Australian Bureau of Statistics. Estimates of Aboriginal and Torres Strait Islander Australians, June 2016. 2018.

[26] Australian Bureau of Statistics. Australian Census 2016 [Available from: https://www.abs.gov.au/websitedbs/D3310114.nsf/Home/Census?OpenDocument&ref=topBar.

[27] Sawrikar P, Muir K (2010). The myth of a ‘fair go’: Barriers to sport and recreational participation among Indian and other ethnic minority women in Australia. Sport Manag Rev.

[28] Whitehead A, Umeh K, Walsh B, Whittaker E, Cronin C. Back to Netball: Motivations for Participation in a Female-Focused Netball Sport Program. Women Sport Phys Activ J. 2019;27(1):21–9.

[29] Joseph C, Naughton G, Antcliff A (2019). Australian netball injuries in 2016: An overview of insurance data. J Sci Med Sport.

[30] Belcher S, Whatman C, Brughelli M, Borotkanics R (2020). Ten-year nationwide review of netball ankle and knee injuries in New Zealand. J Sci Med Sport.

[31] Ooms L, Veenhof C, Schipper-van Veldhoven N, de Bakker DH (2015). Sporting programs for inactive population groups: factors influencing implementation in the organized sports setting. BMC Sports Sci Med Rehabil.

[32] Staley K, Donaldson A, Randle E, Nicholson M, O’Halloran P, Nelson R, Cameron M. Challenges for sport organisations developing and delivering non-traditional social sport products for insufficiently active populations. Aust NZ J Public Health. 2019;43(4):373–81. 10.1111/1753-6405.12912 31339612

[33] Sam MP, Andrew JC, Gee S (2018). The modernisation of umpire development: Netball New Zealand’s reforms and impacts. Eur Sport Manag Quart.

[34] Koorts H, Eakin E, Estabrooks P, Timperio A, Salmon J, Bauman A (2018). Implementation and scale up of population physical activity interventions for clinical and community settings: the PRACTIS guide. Int J Behav Nutr Phys Act.

